# Comparative Analysis of the ELISIO-HX and Xevonta-Hi Dialyzers in Standard Hemodialysis

**DOI:** 10.3390/life15040596

**Published:** 2025-04-03

**Authors:** Blanca Villacorta Linaza, Mario Román Cabezas, María Cristina Sánchez-Pozo, María Paz Alcaide Lara, Rocío Cabra-Rodríguez, Francisco Javier Toro Prieto

**Affiliations:** 1 Departamento de Nefrología, Hospital Universitario Virgen del Rocío, Av. Manuel Siurot s/n, 41013 Seville, Spain; 2 Departamento de Bioquímica Clínica, Hospital Universitario Virgen del Rocío, Av. Manuel Siurot s/n, 41013 Seville, Spain

**Keywords:** expanded hemodialysis (HDx), medium cutoff (MCO) membranes, high-flux dialyzers, uremic toxin clearance, inflammatory markers, standard hemodialysis

## Abstract

As chronic kidney disease (CKD) prevalence rises, optimizing hemodialysis remains essential. While online hemodiafiltration (OL-HDF) is the gold standard, expanded hemodialysis (HDx), i.e., using high-performance dialyzers in standard hemodialysis, remains the most common clinical practice. Medium cutoff (MCO) membranes aim to enhance middle-molecule removal while preserving protein selectivity, although most studies evaluate them in OL-HDF. To this end, this study aims to compare the Xevonta-Hi (B. Braun), a high-flux (HF) polysulfone dialyzer, and the ELISIO-HX (Nipro), an MCO polyethersulfone dialyzer, in standard hemodialysis. In a prospective, observational study, seven stable patients sequentially received treatment with each dialyzer over four weeks. Pre- and post-dialysis levels of small and middle uremic molecules and inflammatory markers—including procalcitonin, prolactin, serum amyloid A, placental growth factor, interleukin-6, haptoglobin, ceruloplasmin, transferrin, prealbumin, and C-reactive protein—were measured. Both dialyzers demonstrated excellent clearance of small and middle molecules, with no significant differences in efficacy. Albumin and total protein losses remained minimal. Moderate reductions in serum amyloid A, placental growth factor, and interleukin-6 were observed, while no significant reductions occurred in the remaining inflammatory markers. These findings support the safety and effectiveness of both MCO and HF dialyzers in standard hemodialysis.

## 1. Introduction

As the global prevalence of diabetes, obesity, and chronic kidney disease (CKD) continues to rise, the demand for kidney replacement therapy is also increasing [1]. Hemodialysis remains a cornerstone treatment for patients with end-stage CKD, aiming to remove uremic toxins, maintain fluid balance, and correct metabolic imbalances, mitigating systemic inflammation [1]. The efficiency of hemodialysis largely depends on the dialyzer membrane, particularly its material composition and pore size, which determine solute removal selectivity and performance [2,3,4].

Online post-dilution hemodiafiltration (OL-HDF) has demonstrated superior clearance of uremic toxins and improved cardiovascular and overall survival compared to conventional hemodialysis [5,6,7]. However, its widespread implementation is limited by technical and economic constraints, making standard hemodialysis the most commonly used modality up to date [8,9].

In recent years, the development of medium cutoff (MCO) membranes has aimed to optimize conventional dialysis by enhancing the removal of middle molecules [10,11]. Expanded hemodialysis (HDx) with these MCO membranes has been incorporated into routine clinical practice, achieving a clearance performance similar to OL-HDF [12,13].

Among high-performance dialyzers, the Xevonta-Hi (B. Braun) and the ELISIO-HX (Nipro) represent two distinct membrane technologies with different permeability characteristics. The Xevonta-Hi, a high-flux dialyzer, features an Amembris™ polysulfone membrane designed to selectively remove small and some medium-sized molecules while minimizing protein loss [14]. In contrast, the ELISIO-HX incorporates an MCO membrane consisting of Polynephron™ polyethersulfone, which offers a broader pore size range, facilitating the clearance of both small and larger middle molecules [15].

Most comparative studies evaluating these dialyzers have focused on their performance in OL-HDF [16,17]. However, given the limitations in OL-HDF accessibility, understanding their efficacy in standard hemodialysis is crucial. This study aims to compare the Xevonta-Hi and ELISIO-HX dialyzers in conventional hemodialysis by assessing the clearance of uremic toxins across different molecular weight ranges, as well as inflammatory markers including transferrin [18], ceruloplasmin [19], haptoglobin [20], prealbumin [21], C-reactive protein (CRP) [22], interleukin-6 (IL-6) [22], placental growth factor (PLGF) [23], and serum amyloid A [24]. By evaluating these parameters, we seek to clarify the practical implications of membrane design on dialysis efficiency in standard clinical practice when OL-HDF is not a viable option.

## 2. Materials and Methods

### 2.1. Inclusion Criteria

This prospective, observational cohort study evaluated the efficacy of two dialyzers in stable hemodialysis patients. Seven patients undergoing maintenance hemodialysis for more than three months participated in the study. Each patient sequentially received treatment with the Xevonta-High-flux Hi 20 dialyzer (B. Braun, Sinsheim, Germany) and the Elisio-HX 21 dialyzer (Nipro, Osaka, Japan) over two consecutive two-week periods, in standard hemodialysis mode. The inclusion criteria were as follows: age > 18 years, stable hemodialysis for ≥3 months, and thrice-weekly dialysis sessions at our peripheral unit. Demographic variables such as age, sex, and underlying chronic kidney disease (CKD) etiology were recorded. Comorbidities including hypertension (HTN), diabetes mellitus (DM), and the absence of active neoplastic disease were documented (Table 1). The patients included in the study were those who initiated dialysis treatment at our hospital unit before being referred to peripheral dialysis centers based on their place of residence. Patients living closer to the hospital were assigned to our peripheral dialysis unit. It is important to note that the selection process was non-intentional, with patients being assigned to the peripheral unit based on administrative and geographic factors, rather than clinical considerations.

### 2.2. Dialysis Procedure

Hemodialysis was performed using the ARTIS PHYSIO dialysis system (Gambro, Baxter, Deerfield, FL, USA). The dialysate composition used in all patients was standardized as follows: calcium 3 mEq/L, potassium 2 mEq/L, bicarbonate 32 mEq/L, sodium 138 mEq/L, magnesium 1 mEq/L, and glucose 1.5 g/L. The dialysate flow rate was set at 500 mL/min throughout the study. Treatment parameters recorded on the day of analytical measurements included dialyzer type, session duration, heparin dosage, Kt/V, vascular access type, and blood flow rate (Table 2). Table 3 shows the membrane characteristics of the dialyzers used. Heparin anticoagulation consisted of either enoxaparin (20/40 mg; 2000/4000 IU/mL) or sodium heparin (10 mg initial dose; 1000 IU/mL) with 5 mg hourly supplements. No hypersensitivity reactions occurred during the sessions. Blood samples were drawn pre- and post-dialysis to assess the clearance of uremic toxins and inflammatory markers. Molecules measured for clearance evaluation included small water-soluble molecules (<500 Da): urea, creatinine, phosphorus, potassium, sodium, and calcium; middle molecules (500 Da–60 kDa): β2-microglobulin, parathyroid hormone, procalcitonin, and prolactin; inflammatory markers: transferrin, ceruloplasmin, haptoglobin, prealbumin, CRP, IL-6, PLGF, and serum amyloid A; as well as albumin and total protein to assess protein losses in serum.

### 2.3. Laboratory Analysis

Biochemical determinations were performed at the Clinical Biochemistry Laboratory of the Virgen del Rocío University Hospital (Seville, Spain). Plasma samples were collected in lithium-heparin containers (BD Vacutainer^®^, Plymouth, England), and serum samples in separator gel tubes (BD Vacutainer^®^, Plymouth, England). Plasma urea was determined enzymatically measuring absorbance (Alinity c-series, Abbott, Chicago, IL, USA). Plasma creatinine was determined using the kinetic alkaline picrate method (Alinity c-series, Abbott, Chicago, IL, USA). Plasma phosphorus and calcium were quantified using colorimetric methods (Alinity c-series, Abbott, Chicago, IL, USA). Plasma sodium and potassium concentrations were measured through ion-selective potentiometry (Alinity c-series, Abbott, Chicago, United States). Parathyroid hormone and procalcitonin were determined in plasma using immunoassays (Alinity ci-series, Abbott, Chicago, IL, USA). Serum β2-microglobulin, prolactin, transferrin, haptoglobin, prealbumin, and high-sensitivity CRP were assessed using immunoassays (Alinity ci-series, Abbott, Chicago, IL, USA). Serum interleukin-6 (IL-6), and placental growth factor (PLGF) were quantified using electrochemiluminescence immunoassays (Elecsys reagent kits on Cobas e801 analyzer, Roche Diagnostics, Basel, Switzerland). Serum amyloid A and ceruloplasmin were measured by rate nephelometry (BN II Nephelometer, Siemens, Berlin, Germany).

### 2.4. Statistical Analysis

Statistical analysis was performed using Python 3.8, utilizing standard data analysis packages. Data manipulation was conducted with Pandas, and hypothesis testing with Scipy and Statsmodels. The normality of the data was evaluated using the Shapiro–Wilk test. All continuous variables followed normal distributions and were expressed as means and standard deviations. Reduction ratios (RRs%) for each solute of interest were calculated using the formula *RR% =* 1 *− (Cpost/Cpre) ×* 100, where *Cpre* and *Cpost* represent pre- and post-dialysis concentrations, respectively. Comparisons of pre-dialysis levels and comparisons of RRs% between dialyzers were made using the Mann–Whitney U test. Data visualizations were created using Seaborn 0.12.0 and Matplotlib 3.10.1, with statistical significance set at *p* < 0.05 (95% Confidence Interval).

## 3. Results

All dialysis sessions were completed without incidents. All patients were hypertensive, and none had active neoplastic disease. No significant differences were observed in the dialysis parameters, including session duration, heparin dosage, Kt/v, vascular access flow rate, vascular access type, and hemodialysis monitor. The pre-dialysis levels of various molecules for each dialyzer are presented in Table 4. No significant differences were found in pre-dialysis levels between dialyzers.

### 3.1. Small Molecules

**Figure 1:**
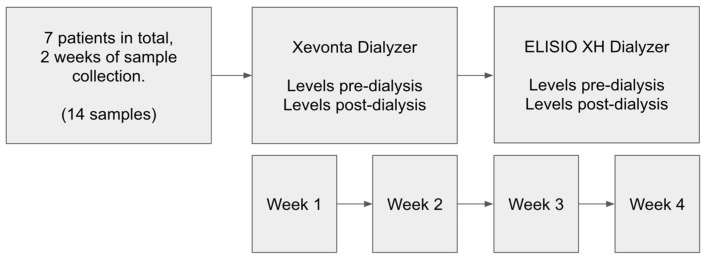
Schematic representation of the study design.

The reduction ratios (RRs%) of small water-soluble molecules (molecular weight < 500 Da), including urea (60 Da), creatinine (113 Da), phosphorus (30 Da), potassium (39 Da), sodium (23 Da), and calcium (40 Da), were calculated for each dialyzer and are shown in Figure 1. High removal capacities of small molecules were demonstrated by both dialyzers, with uremic toxin clearance (urea, creatinine, and phosphorus) ranging from 80% to 60%. The RRs of potassium were approximately 30% in both dialyzers and minimal losses were observed for sodium and calcium. Although the ELISIO-HX dialyzer showed higher removal capacities than the Xevonta-Hi dialyzer across all parameters, these differences were not statistically significant.

### 3.2. Middle Molecules

**Figure 2:**
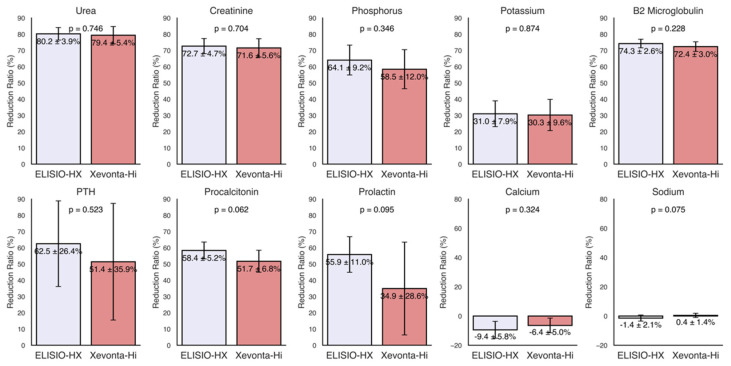
Reduction ratios (RRs%) of small and middle molecules. Small molecules (molecular weight < 500 Da): urea (60 Da), creatinine (113 Da), phosphorus (30 Da), potassium (39 Da), sodium (23 Da), and calcium (40 Da). Middle molecules (molecular weight 500 Da–60 kDa): β2-microglobulin (12 kDa), parathyroid hormone (PTH) (9.4 kDa), procalcitonin (13 kDa), and prolactin (23 kDa). Comparisons of RRs% between dialyzers were made using the Mann–Whitney U test for unpaired samples (95% Confidence Interval).

The RRs% of middle molecules (molecular weight 500 Da–60 kDa), including β2-microglobulin (12 kDa), parathyroid hormone (PTH) (9.4 kDa), procalcitonin (13 kDa), and prolactin (23 kDa), were calculated for each dialyzer and are shown in Figure 2. Both dialyzers exhibited high removal capacities for middle molecules, with RRs% ranging from 75% to 35%. Although the ELISIO-HX dialyzer demonstrated a higher removal capacity than the Xevonta-Hi dialyzer, the differences were not statistically significant.

### 3.3. Inflammatory Markers

**Figure 3:**
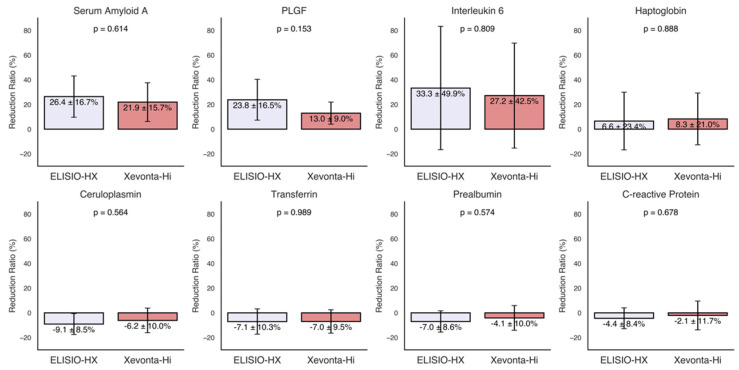
Reduction ratios (RRs%) of inflammatory markers. Inflammatory markers: transferrin, ceruloplasmin, haptoglobin, prealbumin, C-reactive protein, interleukin-6, placental growth factor (PLGF), and serum amyloid A. Comparisons of RRs% between dialyzers were made using the Mann–Whitney U test for unpaired samples (95% Confidence Interval).

The RRs% of various inflammatory markers, including transferrin, ceruloplasmin, haptoglobin, prealbumin, CRP, IL-6, PLGF, and serum amyloid A, were calculated for each dialyzer and are presented in Figure 3. Moderate reductions (30% to 20%) were observed for serum amyloid A, PLGF, and IL-6. The ELISIO-HX dialyzer showed a consistently higher removal capacity than the Xevonta-Hi dialyzer for these inflammatory markers, but the differences were not statistically significant. No significant reductions were observed for the remaining inflammatory markers.

### 3.4. Albumin and Total Protein Loss

Minimal losses were observed in both dialyzers. The mean albumin loss was −257.14 ± 303.35 mg for the ELISIO-HX dialyzer and −164.29 ± 381.57 mg for the Xevonta-Hi dialyzer. The mean total protein loss was −428.57 ± 509.79 mg for the ELISIO-HX dialyzer and −242.86 ± 602.38 mg for the Xevonta-Hi dialyzer. No significant differences in albumin or total protein losses were detected between the two dialyzers.

## 4. Discussion

This study provides a comparative analysis of the ELISIO-HX and Xevonta-Hi dialyzers in standard hemodialysis, focusing on their efficacy in removing small and middle uremic toxins and reducing inflammatory markers. This is one of the few studies that directly compares the performance of these two dialyzers in a real clinical setting, analyzing both solute removal and treatment safety under identical dialysis conditions. Such direct comparisons are fundamental, as dialysis parameters have a marked impact on sieving coefficients and, consequently, on the estimation of RRs% [25].

The results demonstrate that both dialyzers have a remarkable capacity for removing uremic small molecules, with RRs% ranging from 60% to 80% for urea, creatinine, and phosphorus. Minimal losses were found for sodium and calcium, highlighting the favorable safety profile of these dialyzers. Although the ELISIO-HX dialyzer demonstrated superior removal capacity compared to the Xevonta-Hi, the observed differences did not reach statistical significance, demonstrating comparable efficacy between the two dialyzers for the removal of uremic small molecules.

For middle-weight molecules, both dialyzers also demonstrated effective removal capacity, with RRs% values ranging from 35% to 75%. Similarly to small molecules, no significant differences were observed in middle molecule RRs% between the two dialyzers. Notably, RRs% of 70–75% were observed for β2-microglobulin (β2M), a key marker of membrane efficiency. Previous studies have suggested that MCO membranes could surpass high-flux (HF) membranes in β2M clearance in HDx [26,27,28]; however, our results show that, for the duration of the study period, the efficiency of the MCO membrane was non-superior to HF.

An important consideration is the removal of inflammatory markers. Markers such as IL-6 and CRP play a critical role in the persistent inflammatory state observed in hemodialysis patients, contributing to cardiovascular and overall morbidity [29,30]. We found moderate reductions in serum amyloid A, PLGF, and IL-6, with no significant differences between the two dialyzers. This finding suggests that, despite variations in dialyzer structure, the capacity to remove inflammatory mediators is comparable, at least for the duration of the study period.

No reductions were detected in transferrin, ceruloplasmin, haptoglobin, prealbumin, or CRP, which may be explained by their roles as indicators of long-term inflammation rather than acute changes. Longer-term studies are needed to confirm this hypothesis.

Previous randomized controlled trials have shown that MCO membranes can reduce the expression of pro-inflammatory cytokines such as tumor necrosis factor-alpha (TNF-α) and IL-6, although the long-term clinical impact of these reductions remains uncertain [31,32]. Our findings suggest that MCO and HF membranes in HDx may help mitigate the inflammatory burden in hemodialysis patients, potentially improving long-term outcomes.

In terms of safety, both dialyzers demonstrated minimal losses of albumin and total proteins. Specifically, an average albumin loss of −257.14 ± 303.35 mg was recorded for the ELISIO-HX, compared to −164.29 ± 381.57 mg for the Xevonta-Hi. These results are consistent with previous studies [33,34] that independently evaluated each dialyzer, showing that both membranes maintain excellent selectivity, preventing significant losses of essential proteins while enhancing solute clearance.

Altogether, the results indicate comparable performance between the ELISIO-HX and Xevonta-Hi dialyzers in standard hemodialysis, with no significant differences in the removal of small or medium molecules. Despite the theoretical advantage of MCO membranes in enhancing middle-molecule clearance and addressing long-term inflammation, our short-term study did not reveal a measurable benefit over the high-flux dialyzer. These findings suggest that, under standard hemodialysis conditions, the potential advantages of MCO membranes may not translate into immediate differences in uremic toxin removal.

This study presents two primary limitations: a limited sample size and a short-term evaluation period. Increasing the number of participants and extending the duration would provide a more comprehensive understanding of the long-term effects and potential differences between high-flux and MCO membranes. The relatively recent establishment of our dialysis unit has influenced the sample size, and we are actively working to expand the study by including additional patients. Additionally, it may be valuable to explore the performance of these dialyzers under varying dialysis parameters, such as shorter session durations or lower flow rates, which could influence clearance efficiency. It may also be useful to examine hematological parameters and platelet activation markers in future studies to gain a more complete understanding of their potential impact.

A notable strength of this study lies in the standardized comparison of the two dialyzers under identical treatment conditions, ensuring that the results reflect differences inherent to the membranes themselves rather than variations in dialysis settings. Furthermore, the comprehensive assessment of a broad spectrum of molecules—including small and middle uremic toxins, as well as inflammatory markers—provides a thorough evaluation of dialyzer performance across multiple clinically relevant parameters. To further improve this study, its next stage will involve a randomized crossover design with a washout period, maintaining identical treatment conditions between participants. Despite the limitations, the findings demonstrate that, over the short term and within standard hemodialysis conditions, high-flux and MCO membranes perform equivalently. This suggests that both options are suitable for effective uremic toxin removal and inflammation management in routine clinical practice, though longer-term studies are needed to confirm whether these findings persist over time.

## 5. Conclusions

This study highlights the comparable efficacy of the ELISIO-HX and Xevonta-Hi dialyzers in standard hemodialysis. Both dialyzers effectively removed small and medium-weight molecules and maintained a favorable safety profile, with minimal albumin and protein losses. These findings indicate that, within the context of standard hemodialysis, both MCO and HF dialyzers are suitable options for efficient uremic toxin removal and inflammation management. However, we emphasize the need for further studies with larger sample sizes and longer evaluation periods to fully assess their long-term impact on patient outcomes.

## Figures and Tables

**Table 1:** Patient demographic information. Abbreviations used: chronic kidney disease (CKD), diabetes mellitus (DM), hypertension (HT), and active neoplastic disease (ND).

Patient	Age	Sex	CKD Etiology	DM	HT	ND
1	64	M	IgA Nephropathy	Yes	Yes	No
2	55	F	ANCA + Vasculitis	No	Yes	No
3	40	M	Not determined	No	Yes	No
4	54	F	Alport syndrome	No	Yes	No
5	37	F	Not determined	No	Yes	No
6	19	F	Goodpasture syndrome	No	Yes	No
7	67	F	Amyloidosis	No	Yes	No

**Table 2:** Summary of dialysis-related parameters.

Week	Patient	Duration	Heparin Dosage	Kt/v	Vascular Access Flow Rate	Vascular Access Type
1	1	4 h	Enoxaparin 20 mg	1.38	300 mL/min	Arteriovenous Fistula
1	2	3:30 h	Enoxaparin 40 mg	1.64	340 mL/min	Arteriovenous Fistula
1	3	4 h	Enoxaparin 20 mg	1.13	350 mL/min	Central Venous Catheter
1	4	4 h	Enoxaparin 40 mg	1.26	330 mL/min	Prosthetic
1	5	4 h	Enoxaparin 40 mg	1.52	340 mL/min	Central Venous Catheter
1	6	4 h	Enoxaparin 20 mg	1.65	330 mL/min	Central Venous Catheter
1	7	4 h	Sodium heparin 10 mg + 1 × 5 mg	2.04	350 mL/min	Central Venous Catheter
2	1	4 h	Enoxaparin 20 mg	1.53	340 mL/min	Arteriovenous Fistula
2	2	3:30 h	Enoxaparin 40 mg	1.68	340 mL/min	Arteriovenous Fistula
2	3	4 h	Enoxaparin 20 mg	1.19	350 mL/min	Central Venous Catheter
2	4	4 h	Enoxaparin 20 mg	1.26	330 mL/min	Prosthetic
2	5	4 h	Enoxaparin 40 mg	1.63	340 mL/min	Central Venous Catheter
2	6	4 h	Enoxaparin 20 mg	1.65	340 mL/min	Central Venous Catheter
2	7	4 h	Sodium heparin 10 mg + 1 × 5 mg	1.91	350 mL/min	Central Venous Catheter
3	1	4 h	Enoxaparin 20 mg	1.43	340 mL/min	Arteriovenous Fistula
3	2	3:30 h	Enoxaparin 40 mg	1.71	340 mL/min	Arteriovenous Fistula
3	3	4 h	Enoxaparin 20 mg	1.39	340 mL/min	Central Venous Catheter
3	4	4 h	Enoxaparin 20 mg	1.54	340 mL/min	Prosthetic
3	5	3:50 h	Enoxaparin 40 mg	1.62	340 mL/min	Central Venous Catheter
3	6	4 h	Enoxaparin 20 mg	1.65	340 mL/min	Central Venous Catheter
3	7	4 h	Sodium heparin 10 mg + 3 × 5 mg	1.86	340 mL/min	Central Venous Catheter
4	1	4 h	Enoxaparin 20 mg	1.4	300 mL/min	Arteriovenous Fistula
4	2	3:30 h	Enoxaparin 40 mg	1.62	320 mL/min	Arteriovenous Fistula
4	3	4 h	Enoxaparin 20 mg	1.39	340 mL/min	Central Venous Catheter
4	4	4 h	Enoxaparin 20 mg	1.47	320 mL/min	Prosthetic
4	5	4 h	Enoxaparin 40 mg	1.68	340 mL/min	Central Venous Catheter
4	6	4 h	Enoxaparin 20 mg	1.62	340 mL/min	Central Venous Catheter
4	7	4 h	Sodium heparin 10 mg + 1 × 5 mg	1.89	340 mL/min	Central Venous Catheter

**Table 3:** Dialyzer characteristics.

Characteristic	ELISIO-HX 21	Xevonta-Hi 20
Membrane Material	Polynephron™ (Polyethersulfone, BPA-free)	Amembris™ (Polysulfone)
Surface Area (m^2^)	2.1	2.0
Inner Fiber Diameter (µm)	200	195
Membrane Thickness (µm)	40	35
Ultrafiltration Coefficient (mL/h/mmHg)	82	111
Sterilization Method	Dry, oxygen-free gamma	Dry, oxygen-free gamma

**Table 4:** Baseline pre-dialysis levels. Abbreviations used: parathyroid hormone (PTH), placental-like growth factor (PLGF), interleukin-6 (IL-6), C-reactive protein (CRP), not significant (ns).

Parameter	ELISIO-HX Pre Avg ± SD	Xevonta-Hi Pre Avg ± SD	*p*-Value Mann–Whitney’s U test	Interpretation 95% CI
Urea (mg/dL)	118.00 ± 17.00	127.07 ± 28.13	0.6540	ns
Creatinine (mg/dL)	10.14 ± 1.19	10.26 ± 1.12	0.8048	ns
Phosphorus (mg/dL)	4.82 ± 0.93	4.11 ± 1.23	0.3374	ns
Potassium (mEq/L)	4.90 ± 0.57	4.69 ± 0.55	0.5649	ns
Sodium (mEq/L)	136.36 ± 3.08	136.57 ± 2.88	1.0000	ns
Calcium (mg/dL)	9.23 ± 0.32	9.20 ± 0.43	0.9489	ns
B2 Microglobulin (mg/L)	25.74 ± 5.73	26.52 ± 5.64	0.8048	ns
PTH (pg/mL)	751.55 ± 822.07	400.24 ± 186.40	0.5350	ns
Procalcitonin (ng/mL)	0.42 ± 0.26	0.53 ± 0.29	0.4052	ns
Prolactin (ng/mL)	59.58 ± 96.25	42.44 ± 65.91	0.5350	ns
Serum Amyloid A (mg/L)	21.66 ± 30.58	44.31 ± 63.20	0.6200	ns
PLGF (pg/mL)	31.00 ± 7.52	33.51 ± 9.68	0.7104	ns
IL-6 (pg/mL)	13.60 ± 19.09	12.75 ± 19.20	0.6200	ns
Haptoglobin (mg/dL)	110.43 ± 62.21	118.00 ± 68.23	0.9015	ns
Ceruloplasmin (mg/dL)	21.59 ± 5.01	21.13 ± 4.17	0.8478	ns
Transferrin (mg/dL)	181.93 ± 32.94	185.71 ± 36.61	0.9015	ns
Pre-Albumin (mg/dL)	28.66 ± 7.20	29.86 ± 7.03	0.7981	ns
CRP (mg/L)	10.58 ± 15.43	9.69 ± 11.36	1.0000	ns
Albumin (g/dL)	3.81 ± 0.50	3.97 ± 0.57	0.6085	ns
Total Protein (g/dL)	6.23 ± 0.59	6.44 ± 0.68	0.4817	ns

## Data Availability

The data presented in this study are available on request from the corresponding author due to patient confidentiality reasons.

## Abbreviations

The following abbreviations are used in this manuscript:

β2M	β2-Microglobulin
CKD	Chronic kidney disease
CRP	C-reactive protein
HDx	Expanded hemodialysis
IL-6	Interleukin-6
MCO	Medium cutoff
PTH	Parathyroid hormone
PLGF	Placental-like growth factor
OL-HDF	Online hemodiafiltration
